# Supplementary material for: The regulatory landscape of Arabidopsis thaliana roots at single-cell resolution
Source: Nat Commun. 2021 Jun 7;12:3334. doi: 10.1038/s41467-021-23675-y (PMC8184767; doi:10.1038/s41467-021-23675-y)
Supplement: Supplementary file 1 — Supplementary Information [file 41467_2021_23675_MOESM1_ESM.pdf]

Supplementary Information

The regulatory landscape of *Arabidopsis thaliana* roots at single-cell resolution

Dorrity et al.

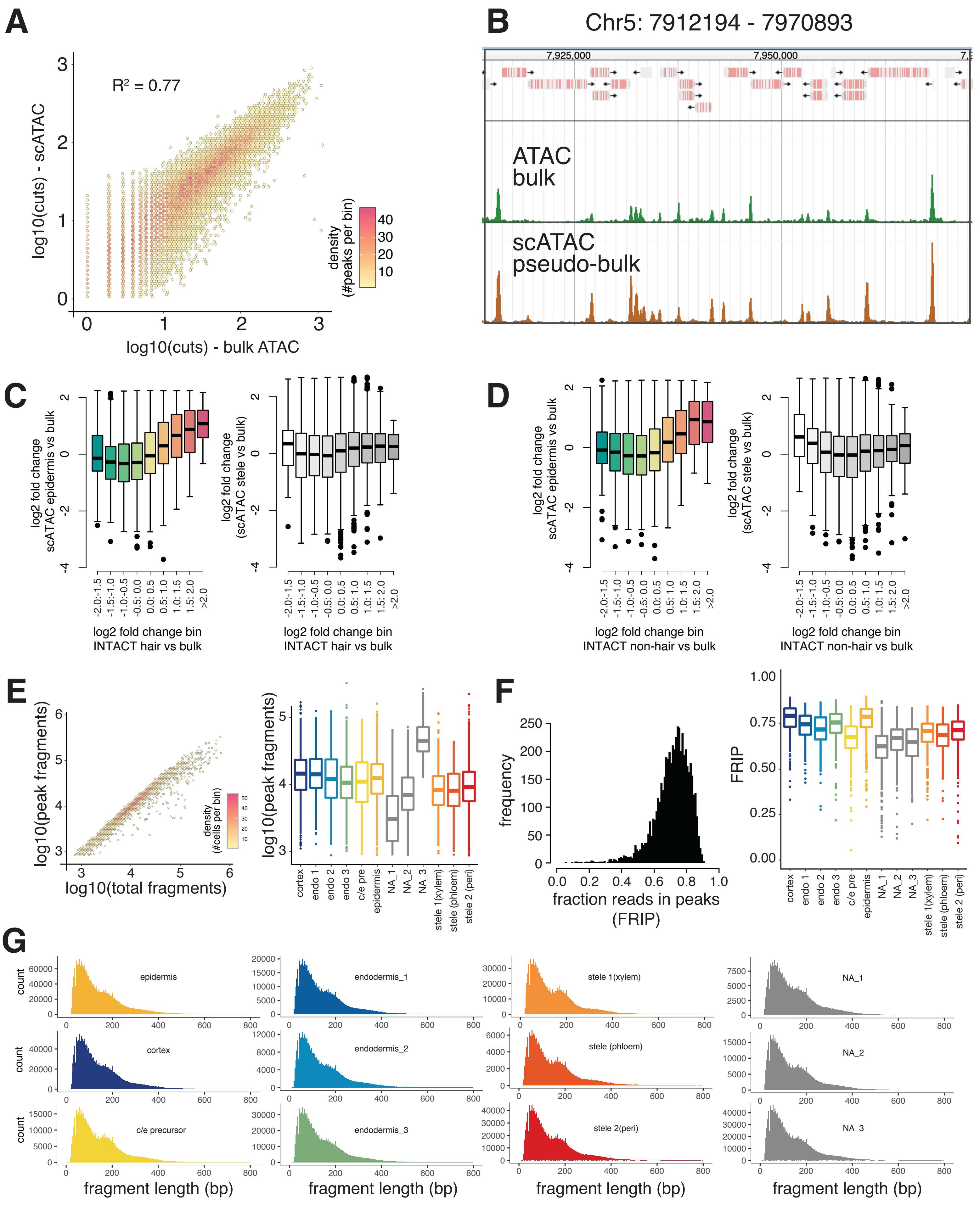

A
R² = 0.77
log10(cuts) - scATAC
log10(cuts) - bulk ATAC
density (#peaks per bin)
B
Chr5: 7912194 - 7970893
7,925,000
7,950,000
ATAC bulk
scATAC pseudo-bulk
C
log2 fold change scATAC epidermis vs bulk
log2 fold change (scATAC stele vs bulk)
log2 fold change bin INTACT hair vs bulk
-2.0:-1.5
-1.5:-1.0
-1.0:-0.5
-0.5: 0.0
0.0: 0.5
0.5: 1.0
1.0: 1.5
1.5: 2.0
>2.0
D
log2 fold change scATAC epidermis vs bulk
log2 fold change (scATAC stele vs bulk)
log2 fold change bin INTACT non-hair vs bulk
E
log10(peak fragments)
log10(total fragments)
density (#cells per bin)
cortex
endo 1
endo 2
endo 3
c/e pre
epidermis
NA_1
NA_2
NA_3
stele 1(xylem)
stele (phloem)
stele 2 (peri)
F
frequency
fraction reads in peaks (FRIP)
FRIP
G
count
epidermis
cortex
c/e precursor
endodermis_1
endodermis_2
endodermis_3
stele 1(xylem)
stele (phloem)
stele 2(peri)
NA_1
NA_2
NA_3
fragment length (bp)

Supplementary Figure 1. Quality of scATAC-seq data is comparable to bulk ATAC-seq data. (A) Scatterplot where each point represents peaks defined in the scATAC data. The x-axis shows the total cutcount within those peaks in bulk ATAC-seq and the y-axis shows the total cutcount within those peaks in scATAC-seq. Point density is indicated by increasing shades of red. (B) Example genomic region showing bulk ATAC accessibility (green) and pseudo-bulked scATAC accessibility (brown). Gene models are indicated above. (C) Boxplots showing peaks from scATAC assay in bins of increasing accessibility from an alternative, cell type-enriched ATAC approach;[11] peaks with low root hair cell-specific accessibility are in the leftmost bin, while those with the greatest root hair cell-specific accessibility are in the rightmost bin. Root hair-specific accessibility was defined as peak accessibility in INTACT-derived root hair cells relative to a bulk ATAC sample. The y-axis in the left panel denotes epidermis-specific accessibility determined from the scATAC experiment, defined by the accessibility of those peaks in epidermal cells relative to accessibility when all cell types are grouped (simulating a "bulk" sample). The y-axis in the right panel denotes the relative accessibility in stele cells as a control. Boxplots are generated using values from individual peaks of in epidermis cells (for each bin, from left to right, n =120, 406, 1166, 2612, 2976, 1532, 519, 162, 70). (D) Identical to (C), except that peaks are grouped by relative accessibility in root non-hair cells, determined by an alternative cell type-enriched ATAC approach.[11](E) Read recovery per cell: Left panel shows relationship between total reads recovered per cell (x-axis) and reads in peaks (y-axis). Areas with higher point density are shown as in (A). Right panel shows boxplots of total number of reads in peaks recovered for each cell type. (F) ATAC quality per cell: Left panel shows the overall distribution of fraction of reads in peaks (FRIP) across all cells, right panel shows distribution of FRIP scores for each cell type. (G) Read length distributions for all fragments separated by cell type.

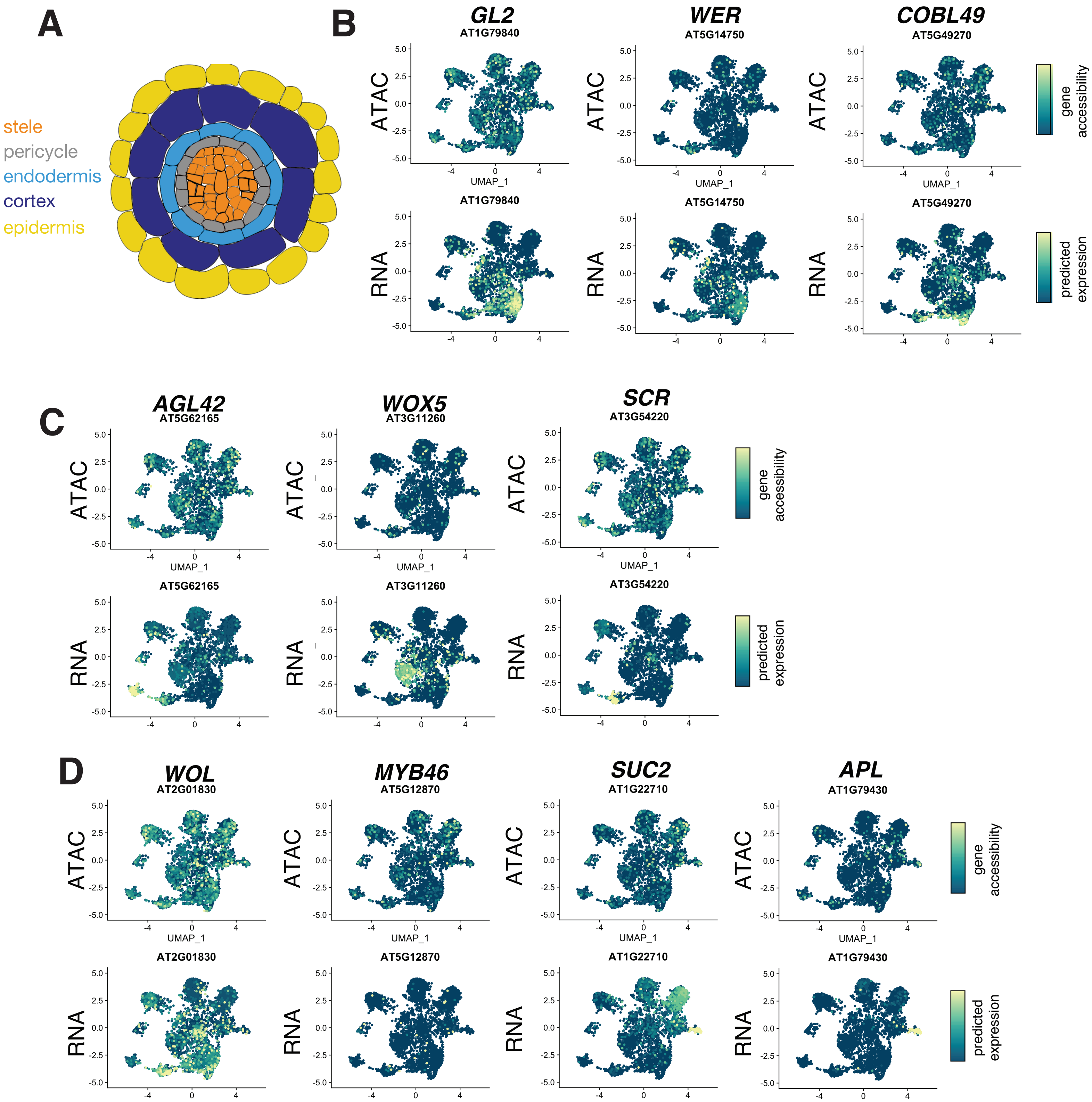

A
stele
pericycle
endodermis
cortex
epidermis
B
GL2
AT1G79840
WER
AT5G14750
COBL49
AT5G49270
ATAC
RNA
UMAP_1
gene accessibility
predicted expression
C
AGL42
AT5G62165
WOX5
AT3G11260
SCR
AT3G54220
D
WOL
AT2G01830
MYB46
AT5G12870
SUC2
AT1G22710
APL
AT1G79430

Supplementary Figure 2. Accessibility and predicted expression levels of traditional marker genes in major cell layers of the root. (A) Schematic showing the major cell layers of the *Arabidopsis* root, colored as in Figure 1. (B) Marker gene plots for epidermis-specific genes showing accessibility (top) and predicted expression levels (bottom). Common and systematic gene names are indicated above. (C) As in previous panel, showing QC (*AGL42*), cortex (*WOX5*), and endodermis (*SCR*) markers. (D) As in previous panels, showing stele (*WOL* and *MYB46*) and phloem (*SUC2* and *APL*) markers.

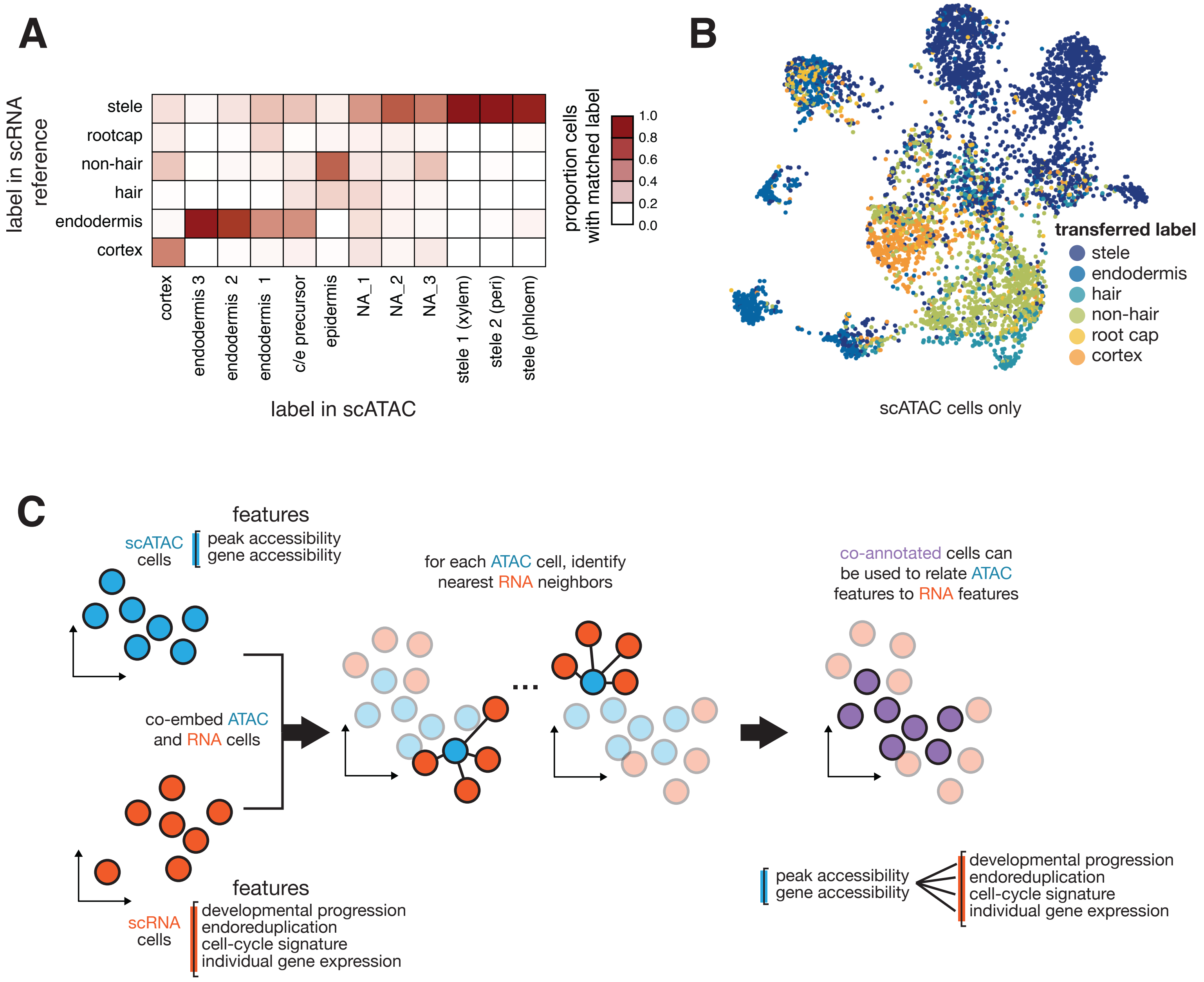
A
label in scRNA reference
stele
rootcap
non-hair
hair
endodermis
cortex
cortex
endodermis 3
endodermis 2
endodermis 1
c/e precursor
epidermis
NA_1
NA_2
NA_3
stele 1 (xylem)
stele 2 (peri)
stele (phloem)
label in scATAC
proportion cells with matched label
1.0
0.8
0.6
0.4
0.2
0.0
B
transferred label
stele
endodermis
hair
non-hair
root cap
cortex
scATAC cells only
C
features
scATAC cells
peak accessibility
gene accessibility
co-embed ATAC and RNA cells
for each ATAC cell, identify nearest RNA neighbors
...
co-annotated cells can be used to relate ATAC features to RNA features
features
scRNA cells
developmental progression
endoreduplication
cell-cycle signature
individual gene expression
peak accessibility
gene accessibility
developmental progression
endoreduplication
cell-cycle signature
individual gene expression

Supplementary Figure 3. Co-embedding of scATAC and scRNA data allows validation of cell type labels and annotation by scRNA-derived features. (A) Confusion matrix showing the correspondence of manual cell annotations (x-axis) with those derived from the label-transfer from RNA to ATAC cells (y-axis). (B) UMAP of scATAC cells as in Fig. 1A, but cells are colored by the cell type label predicted from annotations of scRNA nearest neighbors. These cell type labels broadly match those predicted by manual annotation, and separate the epidermis cluster into hair and non-hair cells. (C) Workflow schematic for annotation of scATAC-cells with transcriptional data. The 25 nearest RNA neighbors from each ATAC cell in the co-embedded graph (**Figure 2A**) were identified, and average expression of individual genes and signatures scores were computed and assigned to each scATAC cell.

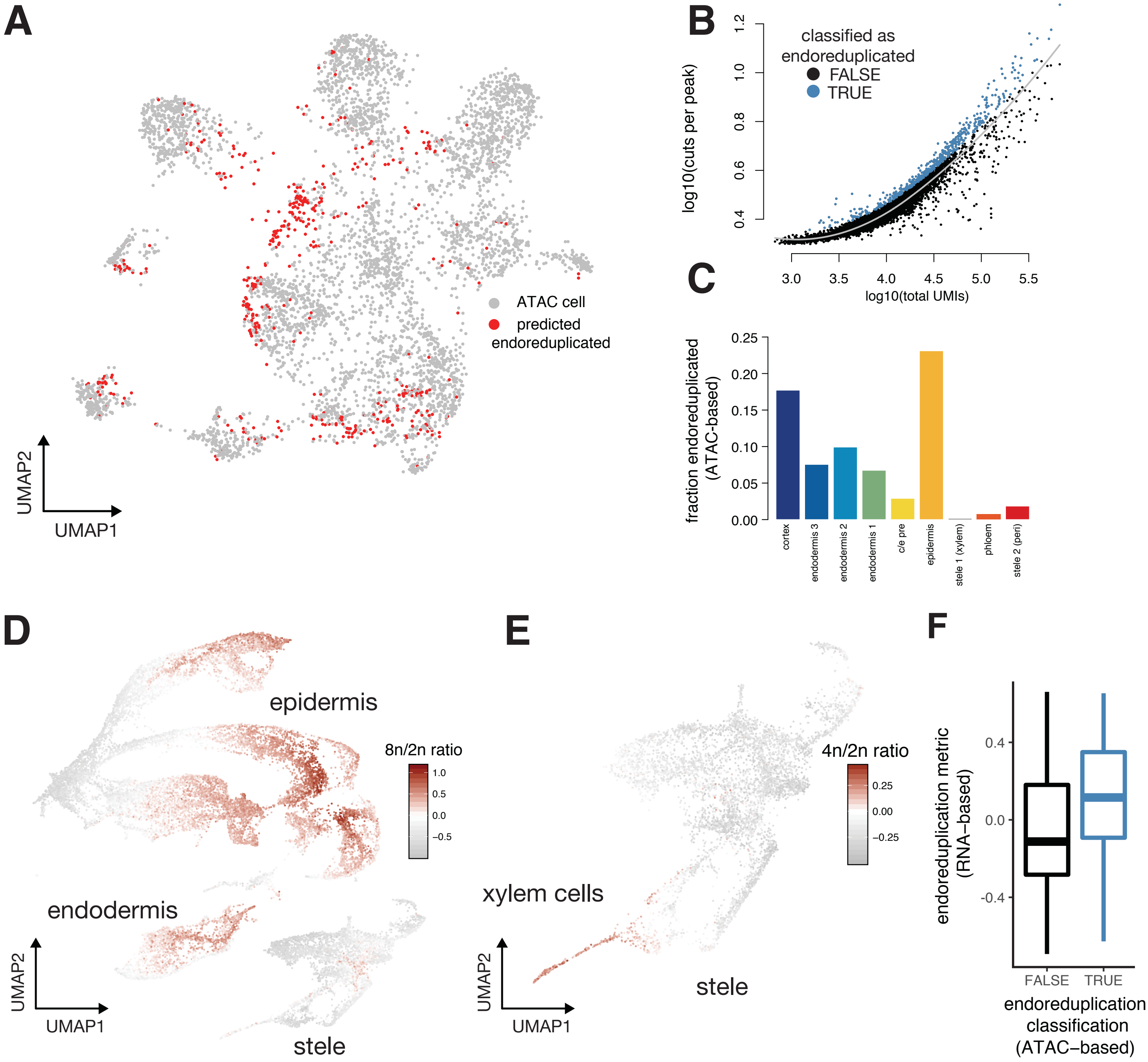
A
ATAC cell
predicted
endoreduplicated
UMAP2
UMAP1
B
classified as
endoreduplicated
FALSE
TRUE
log10(cuts per peak)
log10(total UMIs)
C
fraction endoreduplicated
(ATAC-based)
cortex
endodermis 3
endodermis 2
endodermis 1
c/e pre
epidermis
stele 1 (xylem)
phloem
stele 2 (peri)
D
epidermis
8n/2n ratio
endodermis
stele
E
4n/2n ratio
xylem cells
stele
F
endoreduplication metric
(RNA–based)
FALSE
TRUE
endoreduplication
classification
(ATAC–based)

Supplementary Figure 4. Approaches for identifying endoreduplicated cells in both scATAC and scRNA-seq data. (A) UMAP plot of root scATAC cells, each colored based on whether that cell surpasses a threshold level of cuts per site. Red denotes cells predicted as having undergone endoreduplication. (B) Scatterplot showing the relationship between total UMIs per cell (x-axis) and cuts per peak (y-axis); this relationship was captured in a Loess fit (black line), which was used to determine a threshold for cells with higher cuts per peak than expected based on their total UMIs (cells colored in blue, see Methods for more detail). (C) Barplot showing the fraction of cells in each type that showed putative endoreduplication, as determined by the threshold drawn in (B). In general, outer cell layers showed higher fractions of endoreduplicated cells, while cell layers of the stele showed lower levels. (D) UMAP of root scRNA cells, each colored based on the expression level of a transcriptional signature for endoreduplication, as determined by a ratio of expression levels in genes previously determined as enriched in 8n cells over those enriched in 2n cells.[19] (E) A known instance of endoreduplication in the stele, tetraploid xylem[19], is identified by a metric similar to (D), except that cells are colored by signature for 4n cells (ratio of 4n-specific genes to 2n-specific genes). (F) Boxplot showing the transcriptional-signature-based endoreduplication metric compared to a binary classification of endoreduplication cells using scATAC data. scATAC cells with high levels of cutcounts at a single locus (suggesting endoreduplication, as in A-C) were analyzed in the co-embedded graph with scRNA-seq cells to calculate the average level of the endoreduplication signature among each scATAC cell's 25 nearest neighbors. The overall trend shows that the cutcount-based classification of endoreduplication is consistent with the transcriptional-signature-based metric (n = 4757 FALSE, n = 526 TRUE; one-sided student's t-test $p < 1E^{-14}$).

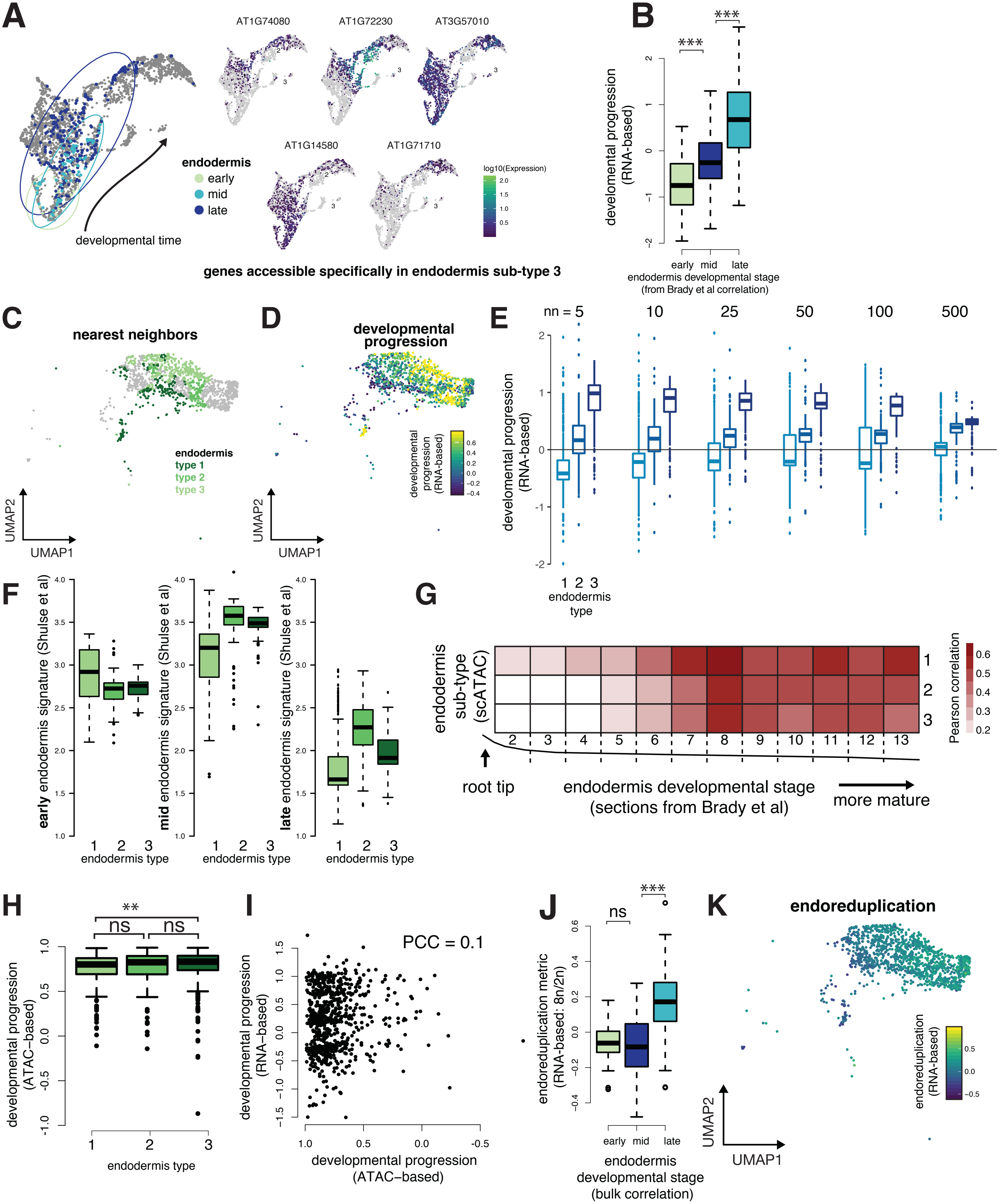
A
AT1G74080
AT1G72230
AT3G57010
AT1G14580
AT1G71710
endodermis
early
mid
late
developmental time
log10(Expression)
genes accessible specifically in endodermis sub-type 3
B
***
***
delevomental progression (RNA-based)
early mid late
endodermis developmental stage
(from Brady et al correlation)
C
nearest neighbors
endodermis
type 1
type 2
type 3
UMAP2
UMAP1
D
developmental progression
developmental progression (RNA-based)
UMAP2
UMAP1
E
nn = 5
10
25
50
100
500
delevomental progression (RNA-based)
1 2 3
endodermis type
F
early endodermis signature (Shulse et al)
mid endodermis signature (Shulse et al)
late endodermis signature (Shulse et al)
1 2 3
endodermis type
G
endodermis sub-type (scATAC)
Pearson correlation
root tip
endodermis developmental stage
(sections from Brady et al)
more mature
H
**
ns
ns
developmental progression (ATAC–based)
endodermis type
I
PCC = 0.1
developmental progression (RNA–based)
developmental progression (ATAC–based)
J
ns
***
endoreduplication metric (RNA-based: 8n/2n)
early mid late
endodermis developmental stage (bulk correlation)
K
endoreduplication
endoreduplication (RNA-based)
UMAP2
UMAP1

Supplementary Figure 5. Characterization of endodermal sub-types with combined scATAC and scRNA-seq data. (A) UMAP of endodermal cells from multiple scRNA-seq studies, with previously-determined developmental stages highlighted.[2] Inset shows variable expression patterns of genes with accessibility patterns specific to endodermal sub-type 3 in the scATAC data. (B) Boxplot showing that developmental progression scores are consistent with previously described annotations of developmental progression (early, middle, late) of the endodermis (all comparisons significant in one-sided students t-test p <1E^{-14}).[2] Cells with highest correlations to "late" endodermis cells (slices 9 – 12 from Brady et al, n = 263) had much greater developmental progression scores than those with highest correlations to "early" endodermis cells (slices 1 – 4, n = 34); cells with highest correlations to "mid" epidermis cells (slices 5 – 8, n = 76) showed an intermediate developmental progression score. (C) Subset of co-embedded UMAP from Figure 2A showing only endodermal cells; 25 nearest RNA neighbors for each endodermal type are indicated in shades of green. (D) As in (C), but shows RNA neighbor cells colored by transcription-based developmental progression metric. (E) Boxplots showing data from Figure 3C, with average developmental progression computed with different numbers of nearest neighbors. Above each plot, the number of neighboring cells (nn) from the scRNA-seq data used to predict developmental progression of each scATAC endodermal cell is shown. The relative differences in predicted developmental progression is insensitive to the number of nearest neighbors used in the procedure. (F) Signature scores computed from early (n = 306 genes), mid (n = 358 genes), and late (n = 134 genes) from a previous scRNA-seq study analyzing endodermis development.[3] Predicted expression of the early signature was highest in endodermis sub-type 1; the middle signature was highest in endodermis sub-type 2; the late signature was also highest in endodermis sub-type 2, but was lowest in endodermis sub-type 1. (G) Heatmap showing Pearson correlation coefficients from each endodermis sub-type's (rows) predicted expression to each stage of endodermis cells defined by FACS-sorted cells (columns along x-axis, from early to late).[1] While all endodermis sub-types appeared to show greatest correlation to endodermis slice 8, the highest correlations for the earliest FACS-sorted endodermis stages was within endodermis sub-type 1. (H) Boxplots showing levels of accessible genes (analogous to transcriptional complexity metric from Fig. 3C, only computed as total number of accessible genes rather than total number of transcribed genes). The overall trend remained the same, with progressive loss of complexity in the later endodermal types (significant for sub-type 1 vs 3, one-sided student's t-test p-value = 0.0032, not significant for other comparisons), but the ATAC-based metric showed less sensitivity than the RNA-based one (cells in early n = 489 cells, mid n = 141 cells, late n = 225 cells). (I) Scatterplot showing poor correlation (PCC = Pearson correlation coefficient) of ATAC-based developmental progression score and the RNA-based score. (J) Boxplot as in (B), showing transcription-based endoreduplication scores (y-axis) for cells annotated for endodermal developmental stages by a previous scRNA-seq experiment (number of cells, n, identical to panel (B), early and middle comparison not significant [ns], middle and late comparison, one-sided students t-test p <1E^{-14}). (K) As in (D), with RNA neighbor cells colored by transcription-based endoreduplication metric.

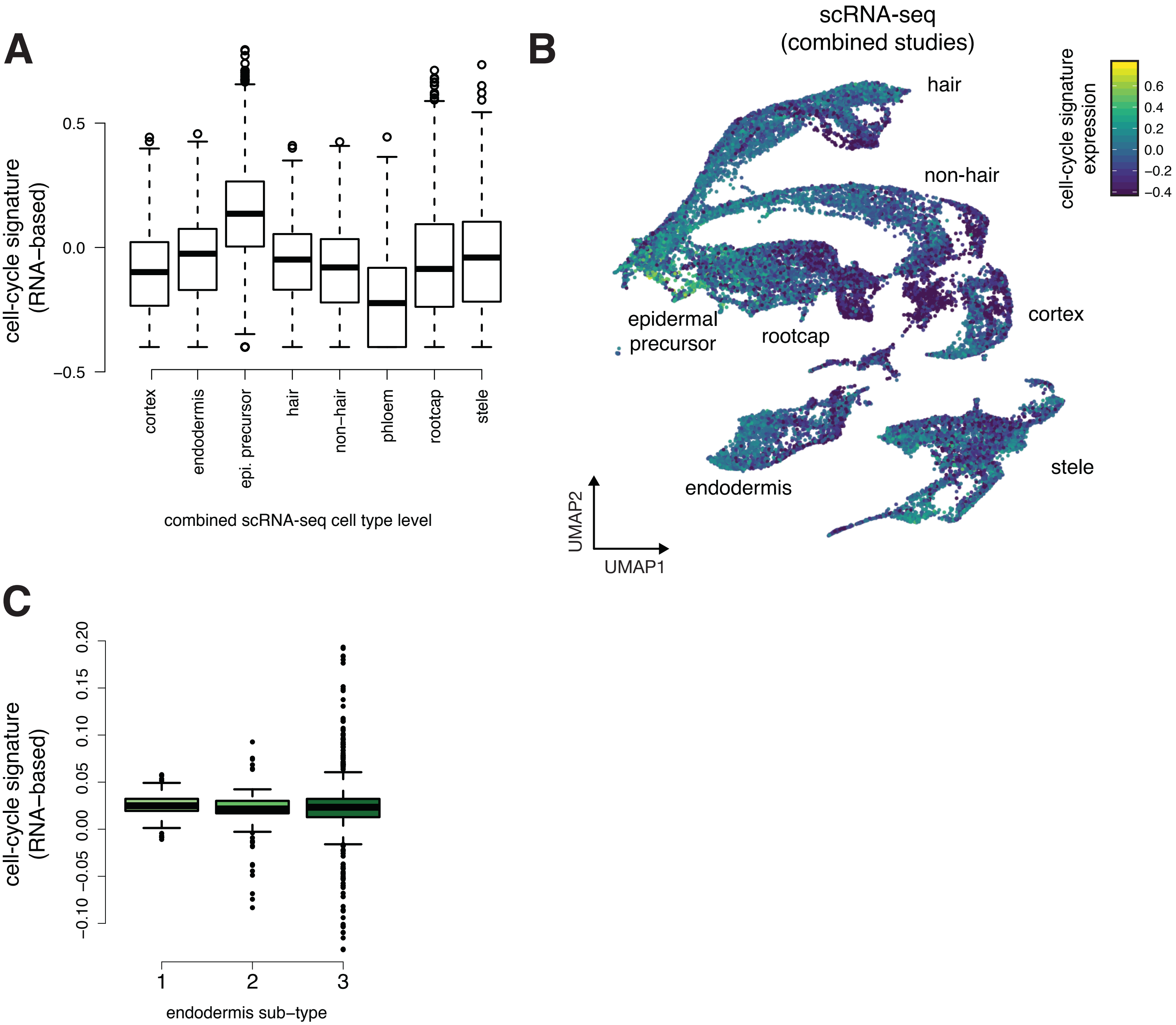
A
cell-cycle signature (RNA−based)
0.5
0.0
−0.5
cortex
endodermis
epi. precursor
hair
non−hair
phloem
rootcap
stele
combined scRNA-seq cell type level
B
scRNA-seq (combined studies)
cell-cycle signature expression
0.6
0.4
0.2
0.0
−0.2
−0.4
hair
non-hair
epidermal precursor
rootcap
cortex
endodermis
stele
UMAP2
UMAP1
C
cell-cycle signature (RNA−based)
0.20
0.15
0.10
0.05
0.00
−0.05
−0.10
1
2
3
endodermis sub−type

Supplementary Figure 6. Dividing cells are present in the root, but do not distinguish endodermis types. (A) Boxplots showing levels of a cell-cycle signature in each scRNA-seq root cell type. (B) UMAP plot of combined root scRNA-seq studies with each cell (cortex n = 1991, endodermis n = 2963, epi precursor n = 1911, hair n = 4185, non-hair n = 3459, phloem n = 535, rootcap n = 5813, stele n = 6181) colored by its expression the cell-cycle signature.[32] (C) Cell-cycle signature predicted from nearest neighbors of endodermis types (as in **Figure 3C, 3D**) shows that proliferation is not a strongly distinguishing feature between the sub-types (cells in early n = 489 cells, mid n = 141 cells, late n = 225 cells).

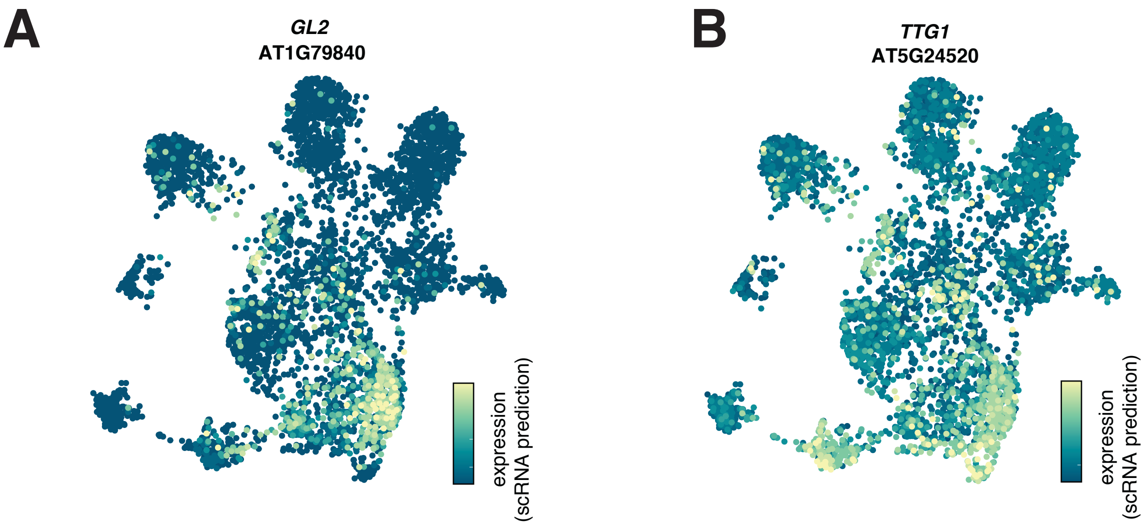
A
GL2
AT1G79840
expression
(scRNA prediction)
B
TTG1
AT5G24520
expression
(scRNA prediction)

Supplementary Figure 7. Identifying transcription factors involved in tissue specification. (A) UMAP of scATAC cells colored by predicted expression level of epidermal specification factor *GL2.* (B) UMAP of scATAC cells colored by predicted expression level of epidermal specification factor *TTG1*.
