# Supplementary material for: The regulatory landscape of Arabidopsis thaliana roots at single-cell resolution
Source: Nat Commun. 2021 Jun 7;12:3334. doi: 10.1038/s41467-021-23675-y (PMC8184767; doi:10.1038/s41467-021-23675-y)
Supplement: Supplementary file 10 — Description of additional supplementary files [file 41467_2021_23675_MOESM10_ESM.pdf]

Description of additional supplementary files

Title: Supplementary Data 1
Description: Marker peaks identified in each cluster.

Title: Supplementary Data 2
Description: Marker genes identified in each cluster.

Title: Supplementary Data 3
Description: Marker genes showing both cell type-specific accessibility (scATAC-seq) and gene expression (scRNA-seq) of root.

Title: Supplementary Data 4
Description: Marker genes identified in endodermis sub-type 1, using expression levels predicted from nearest scRNA-seq neighbors in co-embedding.

Title: Supplementary Data 5
Description: Marker genes identified in endodermis sub-type 2, using expression levels predicted from nearest scRNA-seq neighbors in co-embedding.

Title: Supplementary Data 6
Description: Marker genes identified in endodermis sub-type 3, using expression levels predicted from nearest scRNA-seq neighbors in co-embedding.

Title: Supplementary Data 7
Description: Peaks significantly associated with *TTG2* expression level, derived from linear regression of *TTG2* expression levels with accessibility levels of each peak in epidermis cells.
